# *Toxoplasma gondii* GRA12 Inhibits the NF-ΚB Signaling Pathway by Targeting P65 and the IKK Complex

**DOI:** 10.3390/genes17040476

**Published:** 2026-04-17

**Authors:** Meiling Ou, Xiaowen Fang, Ying Yuan, Zhizhuo Huang, Boren Bai, Xiuying Hou, Yongjun Li, Chunxia Jing, Guang Yang

**Affiliations:** 1 Department of Pathogen Biology, School of Medicine, Jinan University, Guangzhou 510632, China; oumeiling1214@outlook.com (M.O.); xw202505@163.com (X.F.); 13697953357@163.com (Y.Y.); sparrowh@163.com (Z.H.); bai0127@stu2021.jnu.edu.cn (B.B.); houxiuying@jnu.edu.cn (X.H.); yongjunli@jnu.edu.cn (Y.L.); 2 Jiangmen Center for Disease Control and Prevention, Jiangmen 529000, China; 3 Department of Epidemiology, School of Medicine, Jinan University, Guangzhou 510632, China; jcxphd@gmail.com; 4 Guangdong Key Laboratory of Environmental Pollution and Health, Jinan University, Guangzhou 510632, China

**Keywords:** *Toxoplasma gondii*, dense granule protein, GRA12, NF-κB signaling pathway

## Abstract

Background: The NF-κB signaling pathway plays a critical role in innate immune defense against infections. However, many pathogens secrete toxins or effectors into host cells to manipulate cellular functions for their survival and proliferation. *Toxoplasma gondii* is known to establish chronic infections by employing sophisticated immune evasion strategies. Dense granule (GRA) proteins are essential for the survival and pathogenesis of *T. gondii*. Methods: In this study, plasmid transfection, cell culture, luciferase reporter assay, quantitative PCR, and western blot were employed to identify *T. gondii* GRA proteins that regulate the NF-κB pathway. Results: We demonstrate that GRA12, a specific GRA protein, significantly inhibits NF-κB promoter activity and the transcriptional expression of key cytokines, including IL-6, IL-12, TNF-α, and IFN-β. Western blot analysis further revealed that GRA12 suppresses the activation of the IKK complex and p65. Moreover, GRA12 prevents the nuclear translocation of p65. Conclusions: Our findings demonstrate that GRA12 is involved in immune evasion by inhibiting the NF-κB pathway, thereby facilitating *T. gondii* dissemination and infection.

## 1. Introduction

The innate immune response of the host serves as the primary defense mechanism against invading pathogens. [1]. Within the innate immune system, the NF-κB signaling pathway plays a pivotal role in regulating immune responses [2,3]. Consequently, the precise regulation of NF-κB activity has been recognized as a therapeutic target for inflammatory diseases and cancer [4]. An increasing number of studies have highlighted that many intracellular pathogens actively manipulate NF-κB activity to promote their own survival. *Toxoplasma gondii*, in particular, secretes a series of effector proteins that either activate or suppress NF-κB signaling in a strain and context-dependent manner. Typically, NF-κB activation is triggered by pattern-recognition receptors (PRRs) or pro-inflammatory cytokines, including IL-1 and TNF-α [5]. This activation cascade initiates with the recruitment MyD88 and TRADD, which subsequently act on TRAF6 or TRAF2. Subsequently, these intermediates induce activation of the IκB kinase (IKK) complex [6,7,8]. The IKK complex further phosphorylates IκB proteins, targeting them for proteasomal degradation. This releases the NF-κB heterodimer (p65/p50), which then moves to the nucleus to activate target gene transcription. Extensive work has characterized the functional roles of distinct IKK subunits in this cascade: studies [6,7] delineated the scaffolding function of IKKγ in mediating IKK complex assembly; work from [8] identified IKKβ phosphorylation sites that are critical for triggering IκBα degradation; reference [9] characterized the kinetic profile of IKK activation induced by TNF-α stimulation; [10] established that NEMO mutations are associated with impaired NF-κB signaling; and [11] uncovered IKK-dependent post-translational modifications of the p65 subunit that modulate pathway activity.

*T. gondii* is capable of infecting nearly all warm-blooded animals. While *T. gondii* infection is generally asymptomatic in immunocompetent individuals [12], it may pose a life-threatening risk to those with compromised immune function [13]. Clinical manifestations of toxoplasmosis include myocarditis, ocular toxoplasmosis, encephalitis, hydrocephalus and mental diseases [14]. In pregnant women, infection can lead to severe congenital complications, including abortion, neonatal mortality, hydrocephalus, and central nervous system abnormalities [13]. Despite its public health significance, no vaccine has been developed to prevent human toxoplasmosis. Therefore, elucidating the mechanisms by which *T. gondii* modulates host defense is crucial for the developing effective vaccines and novel therapeutics.

Previous studies have suggested that TLRs recognize *T. gondii* components—including its DNA, RNA, and dense granule proteins—to mediate the production of IL-12 and IFN-β [15,16]. Microarray analyses have further showed that *T. gondii* infection enhances the expression of a few NF-κB-regulated genes [17]. However, Butcher et al. found that intracellular *T. gondii* infection does not always induce NF-κB nuclear localization [18]. This observation is inconsistent with a well-documented discrepancy between in vivo and in vitro phenotypes: while NF-κB activation is readily detectable during in vivo infection, *T. gondii* infection often fails to trigger NF-κB signaling in cultured cell models [18,19,20], implying that the parasite has evolved dedicated mechanisms to suppress this pathway under specific conditions. Collectively, these findings indicate that *T. gondii* can dampen NF-κB activation. Although several dense granule (GRA) and rhoptries organelle protein (ROP) effectors have been reported to modulate host immune signaling, the full repertoire of parasite factors that contribute to immune regulation and evasion remains incompletely defined.

During host cell invasion, *T. gondii* resides in the parasitophorous vacuole (PV) [21]. GRA proteins are released into the parasitophorous vacuole (PV) and its membrane (PVM), where they play essential roles in nutrient acquisition and immune evasion during parasite replication [22]. To date, more than 30 GRA proteins have been identified [23,24]. Recent studies have reported that certain GRAs, such as GRA7 and GRA15, can activate innate immunity [25,26]. For instance, GRA7 induces NF-κB signaling via TRAF6 activation and inducing reactive oxygen species (ROS) production, while GRA15 activates NF-κB in a TRAF6- and IKK-dependent manner. These findings highlight GRA proteins as potential effectors in host–parasite interactions.

In this study, we identified that GRA12 significantly reduces the transcription of pro-inflammatory cytokines by downregulating the NF-κB pathway. Specifically, we observed that GRA12 negatively regulates NF-κB activation during innate immune responses by targeting the p65 and IKK complexes. Our results demonstrate that GRA12 is a key effector of immune evasion during *T. gondii* infection.

## 2. Materials and Methods

### 2.1. Cells, Parasites, Antibodies, Plasmids and Reagents

HEK293T cells (obtained from the American Type Culture Collection, ATCC^®^ CRL-3216™, Manassas, VA, USA), TLR4-293 cells (HEK293 cells stably transfected with human TLR4a, MD2, and CD14 genes; obtained from InvivoGen, #293-htlr4md2cd14, Hong Kong, China), and HFF cells (human foreskin fibroblasts; obtained from the American Type Culture Collection, ATCC^®^ SCRC-1041™) were maintained in DMEM (Thermo Fisher, Shanghai, China) supplemented with 10% fetal bovine serum (FBS; Thermo Fisher), 100 U/mL penicillin, and 100 μg/mL streptomycin in a humidified 5% CO_2_ atmosphere at 37 °C. To ensure good cell condition, subculturing at a 1:4 ratio is typically required every 3 days. The GFP-tagged ME49 strain of *T. gondii* was maintained in HFF cells and passaged every three to four days. To isolate the parasites, infected cells were passed five times through a 26-gauge needle, filtered through a 0.5-μm pore filter (Millipore, Bedford, MA, USA), and centrifuged at 3000× *g* for 5 min. The pelleted parasites were then purified by washing with phosphate-buffered saline (PBS; Thermo Fisher) followed by an additional centrifugation at 3000× *g* for 5 min. Lipopolysaccharide (LPS, L2880) was purchased from Sigma. Lipid-mediated transfections were performed using Lipofectamine 2000 or 3000 (Thermo Fisher). Plasmids were transformed into Escherichia coli (*E. coli*) DH5α competent cells (Takara, Dalian, China) and extracted using the QIAprep Spin Miniprep Kit (Qiagen, Shanghai, China). The TNF-α-luciferase reporter plasmid was obtained from Beyotime Institute of Biotechnology (Shanghai, China). For immunoblotting, the following primary antibodies were used at a 1:1000 dilution: anti-β-actin (A4700, Sigma, Shanghai, China), anti-Flag (F3165, Sigma), anti-phospho-IKKα/β, and anti-IκB-α (Cell Signaling Technology, Shanghai, China). HRP-conjugated anti-rabbit and anti-mouse IgG secondary antibodies (Cell Signaling Technology) were used at a 1:2000 dilution.

### 2.2. Plasmid Construction

Total RNA from the ME49 strain of *T. gondii* was extracted from approximately one flask of infected HFF cells (80–90% lysis) using the Universal RNA Extraction Kit (Takara) according to the manufacturer’s instructions, including on-column DNase I digestion to remove genomic DNA. Complementary DNA (cDNA) was synthesized using the iScript cDNA Synthesis Kit (Bio-Rad, Shanghai, China) and subsequently used as a template for PCR amplification of the GRA12 coding sequence (1311 bp). The forward primer (5′-AGA TCT GAT GAG GGC GAT CGT GGC ATC GA-3′) and the reverse primer (5′-TCT AGA TCA GTT GTG TTT GCT GCC TGC AGA GCC-3′) were designed to introduce BglII and XbaI restriction sites, respectively. Following amplification, the PCR products were digested with FastDigest restriction enzymes (Thermo Fisher) and ligated into the p3×FLAG-CMV-7.1 expression vector (E7533, Sigma), which contains an N-terminal 3×FLAG tag, using T4 DNA Ligase (Thermo Fisher). The sequences of the recombinant plasmids were verified by Sanger sequencing (Thermo Fisher). The reference gene and protein sequences of GRA12 were retrieved from ToxoDB (http://toxodb.org/toxo/, accessed on 15 December 2015), and the presence of a signal peptide was predicted using the SignalP server (http://www.cbs.dtu.dk/services/SignalP/, accessed on 10 January 2016) prior to primer design.

### 2.3. Dual-Luciferase Reporter (Dlr) Assays

The Dual-Luciferase Reporter assay measures firefly luciferase activity driven by a promoter of interest and normalizes it to Renilla luciferase from a co-transfected internal control (pRL-TK), controlling for transfection efficiency. HEK293T and TLR4-293 cells were seeded into 24-well plates at a density of 2 × 105 cells/well and cultured for 24 h. The cells were then transiently co-transfected with 100 ng/well of the luciferase reporter plasmid and 100 ng/well of the pRL-TK Renilla luciferase plasmid (as an internal normalization control) using Lipofectamine 2000 (Thermo Fisher). For HEK293T cells, 50 ng of expression plasmids for MyD88, TRAF2, TRAF6, IKKα, IKKβ, TAK1+TAB1, or p65 were co-transfected as indicated. At 24h post-transfection, cells were harvested for luciferase measurement (for experiments without LPS stimulation). For TLR4-293 cells, at 24h post-transfection, cells were stimulated with LPS (50 ng/mL) for 0, 6, 12, or 24h before harvest. Dual-luciferase activities in the total cell lysates were quantified using the Dual-Luciferase Reporter Assay System (Yuanpinghao Biotech, Beijing, China) according to the manufacturer’s instructions. Crucially, the entire experimental workflow, from plasmid preparation to transfection and luminescence reading, was performed in duplicate or triplicate independently. The data presented are pooled from these three independent experiments and are expressed as the mean ± standard deviation.

### 2.4. Quantitative Real-Time PCR

Total RNA was isolated from HEK293T cells with the Omega total RNA kit (Norcross, GA, USA) and was reverse-transcribed with PrimeScript RT Master Mix kit (Takara). The mRNA of the target genes was quantified by real-time PCR with SYBRGreen PCR Master Mix kit (Takara) and the primers are as follows: for human IFN-β, 5′-CTT GGA TTC CTA CAA AGA AGC AGC-3′ and 5′-TCC TCC TTC TGG AAC TGC TGC A-3′; for human IL-6, 5′-AGA CAG CCA CTC ACC TCT TCA G-3′ and 5′-TTC TGC CAG TGC CTC TTT GCT G-3′, for human β-actin (as an internal control), 5′-CAC CAT TGG CAA TGA GCG GTT C-3′ and 5′-AGG TCT TTG CGG ATG TCC ACG T-3′.

### 2.5. Western Blot

TLR4-293 cells were seeded into 6-well plates at a density of 6 × 10^5^ cells/well and transfected with 2 μg/well of either a GRA12 expression plasmid or an empty vector (EV). Twenty-four hours post-transfection, the cells were treated with LPS for the indicated durations (0, 15, 30 min). Total cellular protein was extracted using lysis buffer supplemented with 1 mM PMSF and 1× protease inhibitor cocktail (Cell Signaling Technology). Nuclear and cytoplasmic fractions were isolated using the Beyotime Nuclear and Cytoplasmic Protein Extraction Kit (P0027, Shanghai, China) according to the manufacturer’s instructions, with centrifugation at 14,000× *g* at 4 °C. Protein samples were solubilized in 4× SDS-PAGE loading buffer (Takara), separated by 10% SDS-PAGE, and electrotransferred onto PVDF membranes (Immobilon-P). Following a blocking step, the membranes were incubated with primary antibodies, washed with Western Wash Buffer, and then incubated with HRP-conjugated secondary antibodies (Cell Signaling Technology; 1:2000) for 1 h at 25 °C. Immunoreactive bands were visualized using the BeyoECL Plus kit (Beyotime), and the optical density of each blot was normalized to that of β-actin. Unless otherwise specified, all reagents for Western blot analysis were purchased from Beyotime Institute of Biotechnology (Shanghai, China).

### 2.6. Statistical Analysis

Statistical analyses were performed using GraphPad Prism 9.5.0 software. The experimental data are presented as the mean ± standard deviation (SD). For comparisons between the two groups, Student’s *t*-test (two-sided, non-paired) was used. For comparisons between multiple dose–response experiments and a single control group, one-way analysis of variance (ANOVA) was performed followed by Dunnett’s post hoc test. For time-course experiments involving two factors, two-way ANOVA was used followed by Tukey’s post hoc test to analyze the simple effects at each time point. Statistical significance is defined as follows: * *p* < 0.05, ** *p* < 0.01, *** *p* < 0.001 and **** *p* < 0.0001.

## 3. Results

### 3.1. Sequence Analysis of GRA12 Cdna and Its Predicted Protein Product

**Figure 1:**
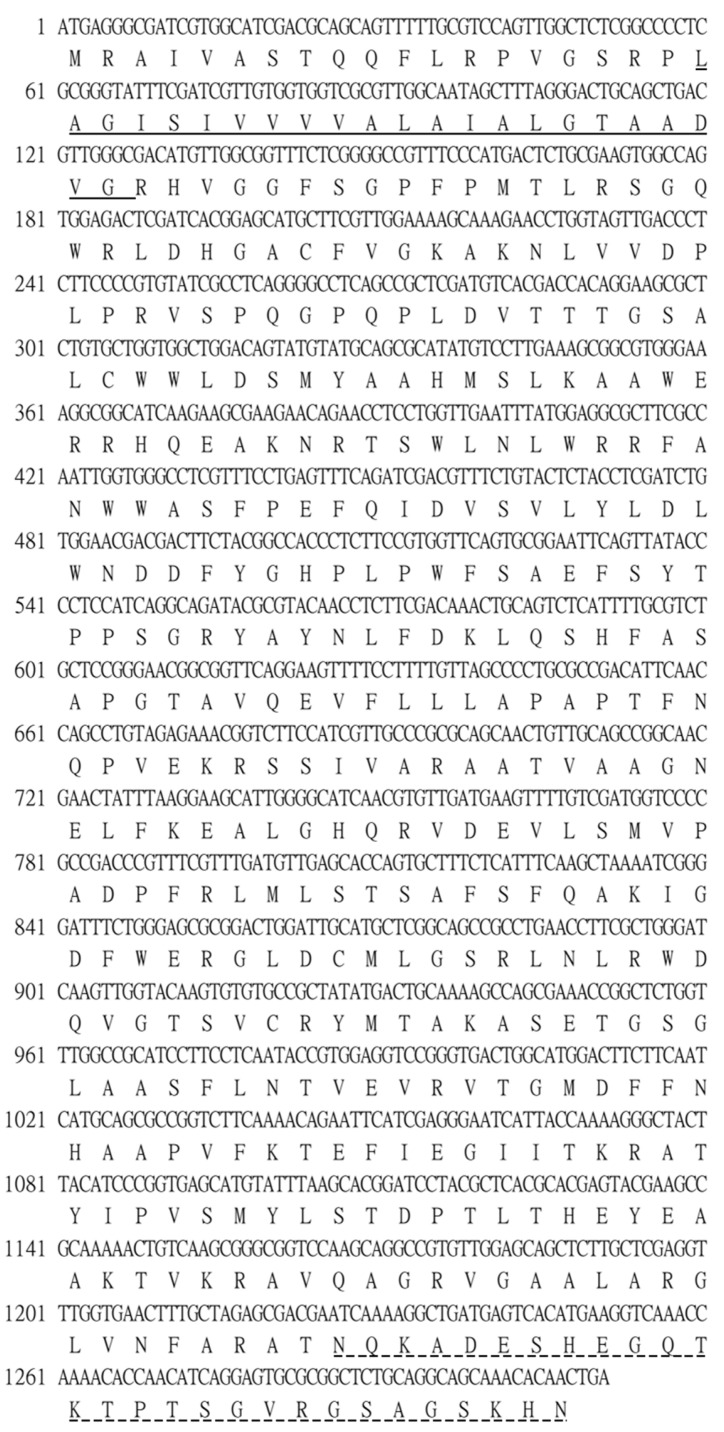
Nucleotide and deduced amino acid sequences of *T. gondii* GRA12. The 1311 bp cDNA sequence is displayed (top line) with its corresponding amino acid sequence (bottom line) shown in single-letter code. Numbers on the left indicate the position of the first nucleotide in each line. The predicted transmembrane domain is indicated by a solid underline, while the MobiDB-lite domain is denoted by a dashed underline. The sequence data have been deposited in GenBank under accession number NC_031477.1.

RNA extraction from HFF cells infected with *T. gondii*, followed by cDNA amplification and cloning of GRA12 mRNA. Bioinformatic analysis of the GRA12 cDNA sequence revealed an 1131-bp open reading frame encoding a putative protein of 436 amino acids, with a predicted molecular mass of 47.9 kDa (Figure 1). Structural characterization identified a transmembrane domain spanning residues 20–42 and a MobiDB-lite domain between residues 408–436. The presence of this transmembrane domain suggests that GRA12 may exist in both soluble and membrane-associated forms, potentially facilitating its functional versatility within the host environment.

### 3.2. GRA12 Inhibited the NF-κB Promoter Luciferase Activity

**Figure 2:**
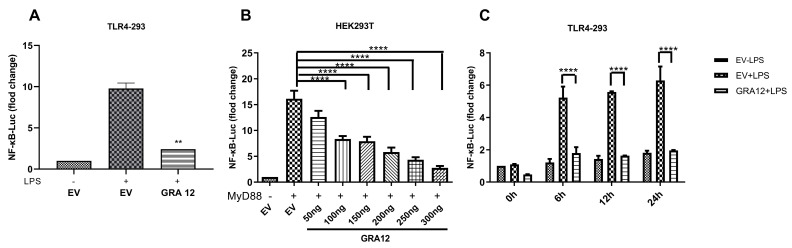
GRA12 negatively regulated NF-κB luciferase activity. For all assays, the relative luciferase activity was normalized to the co-transfected internal control plasmid pRL-TK. (**A**) TLR4-293 cells were co-transfected with the GRA12 expression plasmid (200 ng/well) and the NF-κB luciferase reporter plasmid (100 ng/well). At 24 h post-transfection, cells were stimulated with LPS (50 ng/mL) for 6 h prior to the measurement of luciferase activity. Data are mean ± SD (the sample size is duplicate). Student’s *t*-test (**B**) HEK293T cells were co-transfected with the NF-κB reporter, MyD88 expression plasmid, and increasing amounts of the GRA12 plasmid (50, 100, 150, 200, 250, and 300 ng). Luciferase activity was assessed 24 h post-transfection. Data are mean ± SD (the sample size is triplicate). One-way ANOVA with Dunnett’s post hoc test vs. EV. (**C**) TLR4-293 cells were co-transfected with the GRA12 plasmid (200 ng) and the NF-κB reporter for 24 h, followed by LPS treatment (50 ng/mL) for 0, 6, 12, and 24 h before measuring luciferase activity. Data are mean ± SD (the sample size is duplicate). Two-way ANOVA with Tukey‘s post hoc test. (** *p* < 0.01 and **** *p* < 0.0001. Exact *p*-values are provided in Supplementary Table S1).

To address the contribution of GRA12 to NF-κB signaling, TLR4-293 cells were co-transfected with individual GRA12 plasmids and a luciferase reporter driven by the NF-κB promoter. At 24 h post-transfection, the cells were stimulated with LPS (50 ng/mL) for 6 h, followed by a dual-luciferase reporter (DLR) assay. Among the GRA family members tested, GRA12 was found to significantly inhibit NF-κB luciferase activity (Figure 2A).

To further validate this result, we co-transfected HEK293T cells with MyD88 and varying concentrations of the GRA12 expression plasmid for the DLR assay. As shown in Figure 2B, co-transfecting the empty vector (EV) and MyD88 resulted in an approximately 15-fold induction of NF-κB luciferase activity. However, GRA12 strongly suppressed the MyD88-induced activation of the NF-κB promoter in a dose-dependent manner.

We subsequently examined the time-dependent nature of this inhibition. As shown in Figure 2C, compared to TLR4-293 cells transfected with EV alone, GRA12 significantly suppressed the basal NF-κB luciferase activity even in the absence of LPS stimulation (0 h). Following LPS stimulation to trigger NF-κB activation, GRA12 consistently inhibited the luciferase activity at 6 h, 12 h, and 24 h. Collectively, these results demonstrate that GRA12 negatively regulates the NF-κB promoter activity.

### 3.3. GRA12 Suppresses the Expression of NF-κB-Target Cytokines Il-6, Il-12, Tnf-A, and Ifn-Β

**Figure 3:**
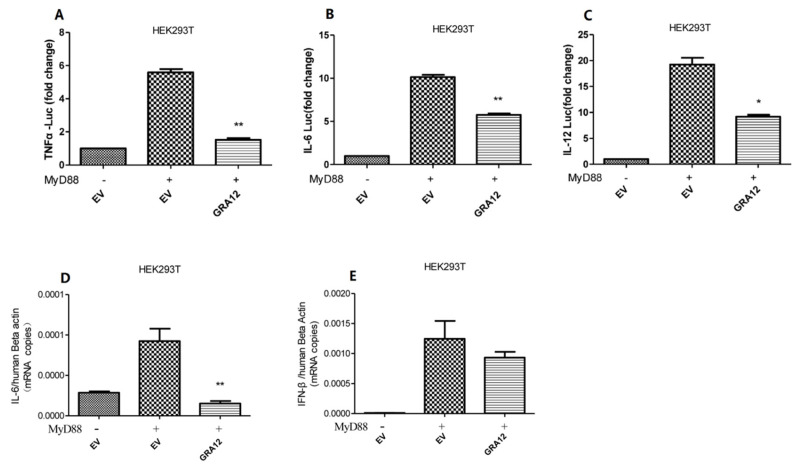
GRA12 negatively regulates the expression of TNF-α, IL-6, IL-12, and IFN-β. (**A**–**C**) HEK293T cells were co-transfected with the GRA12 expression plasmid (200 ng/well) or an empty vector (EV) in the presence or absence of MyD88. At 24 h post-transfection, cells were harvested for dual-luciferase reporter (DLR) analysis of TNF-α, IL-6, and IL-12 promoter activities. For DLR assays, relative luciferase activity was normalized to the co-transfected internal control pRL-TK. Data are mean ± SD (the sample size is duplicate). Student’s *t*-test. (**D**,**E**) HEK293T cells were transfected as described above, and mRNA levels of IL-6 and IFN-β were quantified by qRT-PCR at 24 h post-transfection, with values normalized to β-actin. Data are mean ± SD (the sample size is triplicate). Student’s *t*-test. (* *p* < 0.05, ** *p* < 0.01. Exact *p*-values are provided in Supplementary Table S1).

NF-κB is a pivotal transcription factor that regulates the expression of various downstream effectors, including IL-6, IL-12, TNF-α, and IFN-β. To further validate the inhibitory role of GRA12 in NF-κB signaling, we assessed the promoter activities of TNF-α, IL-6, and IL-12 using dual-luciferase reporter assays. As illustrated in Figure 3A–C, GRA12 significantly attenuated the luciferase activity of all three cytokine reporters. Consistently, qRT-PCR analysis revealed that GRA12 overexpression led to a reduction in the mRNA levels of IL-6 and IFN-β (Figure 3D,E). Collectively, these results indicate that GRA12 effectively inhibits the transcription of key NF-κB-dependent inflammatory genes.

### 3.4. GRA12 Negatively Regulates NF-κB Signaling at the Level of the Ikk Complex and P65

**Figure 4:**
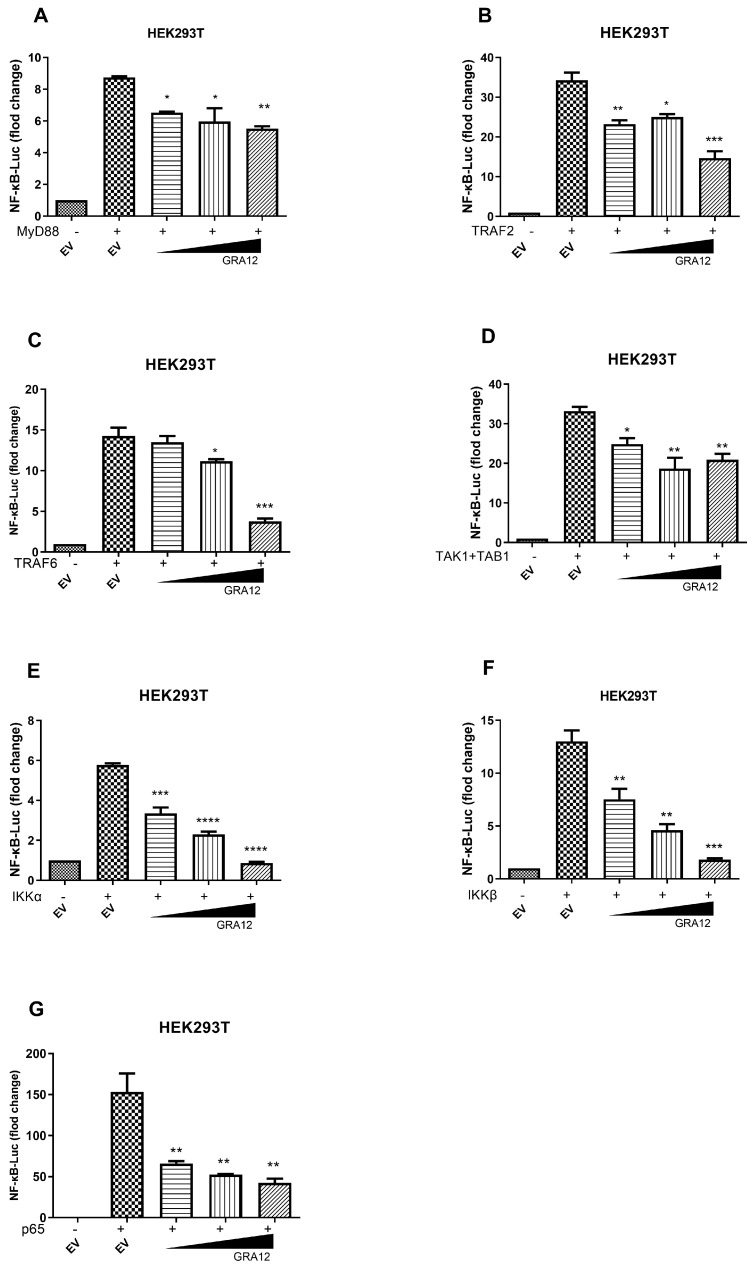
GRA12 inhibits NF-κB activation at or downstream of p65. HEK293T cells were co-transfected with the NF-κB-Luc reporter plasmid, the Renilla luciferase internal control plasmid (pRL-TK), and an expression construct for MyD88 (**A**), TRAF2 (**B**), TRAF6 (**C**), IKKα (**D**), IKKβ (**E**), TAK1+TAB1 (**F**), or p65 (**G**), together with either an empty vector (EV) or the GRA12 expression plasmid. At 24 h post-transfection, cells were harvested and subjected to dual-luciferase reporter (DLR) analysis. The relative luciferase activity for each sample was normalized to the pRL-TK internal control. Data are mean ± SD (the sample size is duplicate). One-way ANOVA with Dunnett‘s post hoc test vs. EV. (* *p* < 0.05, ** *p* < 0.01, *** *p* < 0.001 and **** *p* < 0.0001. Exact *p*-values are provided in Supplementary Table S1).

To further investigate the exact level at which GRA12 negatively regulates NF-κB activation, we performed a dose–response assay by co-transfecting increasing amounts of the GRA12 plasmid along with expression constructs for key NF-κB signaling components, including MyD88, TRAF2, TRAF6, TAK1+TAB1, IKKα, IKKβ, and p65. As shown in Figure 4, overexpression of these individual components resulted in a 6- to 150-fold induction of NF-κB-Luc reporter activity. Importantly, GRA12 markedly inhibited the NF-κB activation driven by all tested components, including MyD88, TRAF2, TRAF6, TAK1+TAB1, IKKα, IKKβ, and p65. These results indicate that GRA12 likely suppresses the NF-κB signaling pathway at or downstream of the p65 level.

### 3.5. GRA12 Inhibits Ikkα/β and P65 Phosphorylation, Blocks Iκbα Degradation, and Represses Lps-Induced Nuclear Translocation of P65

**Figure 5:**
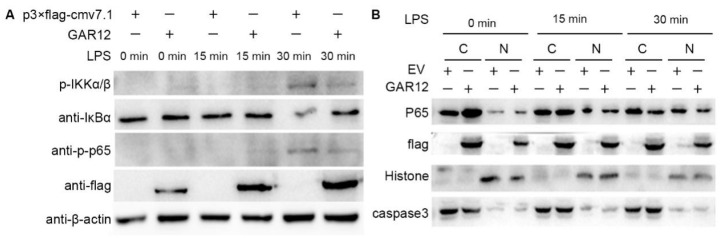
GRA12 inhibits IKKα/β and p65 phosphorylation, blocks IκBα degradation, and represses LPS-induced nuclear translocation of p65. (**A**) TLR4-293 cells were transiently transfected with either a GRA12 expression plasmid (2 μg) or an empty vector (EV) for 24 h. Following transfection, cells were stimulated with LPS (50 ng/mL) for 0 h, 15 min, 30 min. Whole-cell lysates were then subjected to immunoblotting using specific antibodies against phospho-IKKα/β (p-IKKα/β), IκB-α, phospho-p65 (p-p65), Flag, and β-actin (as a loading control). (**B**) TLR4-293 cells were transiently transfected with either the GRA12 expression plasmid (2 μg) or an empty vector (EV) for 24 h. Cells were then stimulated with LPS (50 ng/mL) for 0, 15, 30 min. Cytoplasmic (C) and nuclear (N) fractions were isolated and analyzed by immunoblotting using antibodies against Flag, phospho-p65 (p-p65), Histone H3 (nuclear marker), and Caspase-3 (cytoplasmic marker).

Next, we evaluated the effect of GRA12 on the expression and activation of key pathway proteins by examining the response of GRA12-transfected TLR4-HEK293 cells to LPS stimulation. As indicated in Figure 5A, GRA12 suppressed the phosphorylation of IKKα/β, the degradation of IκBα, and the phosphorylation of p65 at 30 min, suggesting that GRA12 might negatively regulate the NF-κB signaling pathway by targeting the IKK complex.

The nuclear translocation of p65 was examined in TLR4-293 cells following GRA12 overexpression and LPS stimulation using nucleocytoplasmic fractionation and Western blotting. As demonstrated in Figure 5B, Flag-tagged GRA12 itself was detected within the nuclear fraction. Furthermore, p65 translocation into the nucleus was markedly inhibited by GRA12 at 30 min post-LPS stimulation. Collectively, these results suggest that GRA12 translocates into the nucleus, where it potentially interferes with p65-mediated transcriptional activation and the subsequent host immune response.

## 4. Discussion

The NF-κB signaling pathway is a vital component of the innate immune system, playing a central role in regulating genes associated with defense against pathogen infection. Additionally, the NF-κB pathway modulates other cellular processes required for a coordinated immune response, including cell growth, differentiation, adhesion, and survival. Here, we identify GRA12 as a novel *T. gondii* effector that suppresses NF-κB activation through dual targeting of IKK phosphorylation and p65 nuclear translocation. GRA12 differs from well-characterized GRAs such as GRA15 and GRA7 in that it does not activate NF-κB signaling; instead, our data suggest that GRA12 functions as a negative regulator of NF-κB promoter activity. In contrast, GRA15 is known to strongly induce NF-κB activation, while GRA7 modulates host immunity through distinct pathways independent of direct NF-κB inhibition. However, accumulating evidence also reveals that these parasites can directly manipulate the host NF-κB pathway to facilitate immune evasion [27]. For instance, the *T. gondii* effector ROP16 directly phosphorylates the host transcription factor STAT3, an event associated with impaired IL-12 and TNF-α production [28]. Furthermore, ROP18 targets host immunity-related GTPases (IRGs), preventing their accumulation on the parasitophorous vacuole (PV) and thereby promoting parasite survival [29].

**Figure 6:**
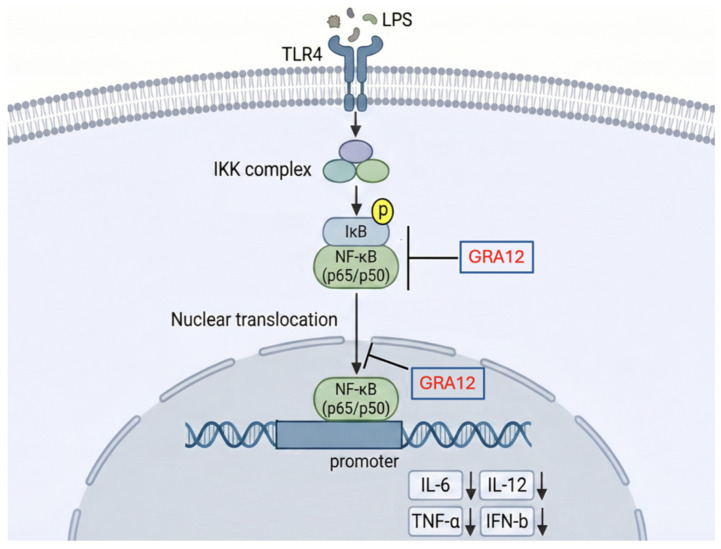
Proposed model for GRA12-mediated inhibition of the host NF-κB signaling pathway.

Dense granule proteins (GRAs) of *T. gondii* are secreted to remodel and maintain the PV, playing critical roles in nutrient uptake and immune evasion [30]. We have found that several GRA family members could negatively regulate NF-κB-driven luciferase activity in the present study with GRA12 exhibiting the most pronounced inhibitory effect. Furthermore, GRA12 significantly suppressed the expression of downstream NF-κB-dependent cytokines, including IL-6, IL-12, and TNF-α (Figure 6). The findings indicate that GRA12, a *T. gondii* dense granule protein associated with the intravacuolar membranous nanotubular network [31], may participate in immune evasion by negatively regulating the host NF-κB signaling pathway. This aligns with emerging evidence that GRAs exhibit functional diversification, though GRA12’s dual-targeting approach contrasts with other GRAs reported to modulate single nodes like MAPK pathways.

Previous studies have shown that *T. gondii* can suppress the NF-κB signaling pathway by interfering with signal transduction cascades, such as attenuating the phosphorylation of p65 [19] and MAPK [32], thereby downregulating the expression of IL-12 and TNF-α. Another study indicated that *T. gondii* heat shock protein 70 might inhibit the NF-κB signaling pathway by targeting the IκB complex [33]. Therefore, to determine the exact level at which GRA12 interferes with this signaling cascade, we overexpressed key pathway components, including MyD88, TRAF2, TRAF6, TAK1+TAB1, IKKα, IKKβ, and p65. We found that the NF-κB activation driven by all of these components was markedly inhibited by GRA12. Consistently, Western blot analysis demonstrated that GRA12 inhibited the phosphorylation of the IKK complex, the subsequent degradation of IκBα, and the phosphorylation of p65. Collectively, these results suggest that GRA12 exerts its negative regulatory effects on the NF-κB pathway via a dual mechanism: targeting the IKK complex upstream and acting at or downstream of p65.

In summary, we characterized GRA12, a *T. gondii* dense granule protein, as a novel negative regulator of the host NF-κB signaling pathway. Notably, we also observed that GRA12 itself can translocate into the host cell nucleus to further inhibit p65 activation. To our knowledge, this is the first study to report that GRA12 participates in *T. gondii* immune evasion by attenuating the NF-κB signaling pathway. By dampening this critical innate immune response, GRA12 may promote the survival and persistence of *T. gondii* within host cells, which requires further investigation using GRA12 gene knockout strains in *T. gondii*-infected cells and animals. These findings elucidate a novel molecular mechanism underlying *T. gondii* immune evasion and provide new insights for future anti-parasitic therapeutic research.

We acknowledge several limitations in the present study. First, our reliance on transient overexpression systems in HEK293T and TLR4-293 cells may not fully recapitulate the physiological context of natural *T. gondii* infection in primary host immune cells. Second, the absence of in vivo validation using GRA12-knockout parasites limits the immediate translational relevance of our findings. Third, the exact molecular mechanisms by which GRA12 targets the IKK complex and p65 remain elusive, warranting further investigation into potential direct protein–protein interactions or specific post-translational modifications. Collectively, we have characterized GRA12 as a novel *T. gondii* immunomodulator that attenuates NF-κB signaling through the dual targeting of IKK phosphorylation and p65 nuclear translocation. While these findings elucidate a critical mechanism underlying parasite immune evasion, future studies utilizing GRA12-knockout strains in both primary cell and animal infection models are required to validate these observations and explore the therapeutic potential of targeting this pathway. Since we did not perform co-immunoprecipitation or binding assays, the conclusions drawn in this study are that “GRA12 inhibits IKKα/β and p65 phosphorylation, blocks IκBα degradation, and represses LPS-induced nuclear translocation of p65.” The specific protein targets directly interacted with by GRA12 remain unclear.

## Data Availability

The original contributions presented in this study are included in the article/Supplementary Materials. Further inquiries can be directed to the corresponding author.

## Supplementary Materials

The following supporting information can be downloaded at: https://www.mdpi.com/article/10.3390/genes17040476/s1, Table S1: Exact *p*-values for Figures.
