# Supplementary material for: Toxoplasma gondii GRA12 Inhibits the NF-ΚB Signaling Pathway by Targeting P65 and the IKK Complex
Source: Genes (Basel). 2026 Apr 17;17(4):476. doi: 10.3390/genes17040476 (PMC13116389; doi:10.3390/genes17040476)
Supplement: Supplementary file 1 [file genes-17-00476-s001.zip › genes-4244946-supplementary.pdf]

Supplementary Table S1. Exact p-values for Figures.

	Comparison	Exact *p*-value	Significance
Figure 2A	EV+ vs. GRA12	0.0041	*
Figure 2B	EV+ vs. GRE12(50ng)	0.0559	ns (no significance)
	EV+ vs. GRE12(100ng)	<0.0001	****
	EV+ vs. GRE12(150ng)	<0.0001	****
	EV+ vs. GRE12(200ng)	<0.0001	****
	EV+ vs. GRE12(250ng)	<0.0001	****
	EV+ vs. GRE12(300ng)	<0.0001	****
Figure 2C	0h: EV+LPS vs. GRA12+LPS	0.4411	ns
	6h: EV+LPS vs. GRA12+LPS	<0.0001	****
	12h: EV+LPS vs. GRA12+LPS	<0.0001	****
	24h: EV+LPS vs. GRA12+LPS	<0.0001	****
Figure 3A	EV+ vs. GRA12 (TNFα)	0.0029	**
Figure 3B	EV+ vs. GRA12 (IL-6)	0.0051	**
Figure 3C	EV+ vs. GRA12 (IL-12)	0.0169	*
Figure 3D	EV+ vs. GRA12 (IL-6 mRNA)	0.0078	**
Figure 3E	EV+ vs. GRA12 (IFN-β mRNA)	0.3765	ns
Figure 4A	MyD88: EV+ vs. GRA12(50ng)	0.0260	*
	MyD88: EV+ vs. GRA12(100ng)	0.0104	*
	MyD88: EV+ vs. GRA12(200ng)	0.0053	**
Figure 4B	TRAF2: EV+ vs. GRA12(50ng)	0.0052	**
	TRAF2: EV+ vs. GRA12(100ng)	0.0111	*
	TRAF2: EV+ vs. GRA12(200ng)	0.0004	***
Figure 4C	TRAF6: EV vs. GRA12(50ng)	0.7673	ns
	TRAF6: EV vs. GRA12(100ng)	0.0396	*
	TRAF6: EV vs. GRA12(150ng)	0.0002	***
Figure 4D	TAK1+TAB1: EV vs. GRA12(50ng)	0.0408	*
	TAK1+TAB1: EV vs. GRA12(100ng)	0.0040	**
	TAK1+TAB1: EV vs. GRA12(200ng)	0.0085	**
Figure 4E	IKKα: EV vs. GRA12GRA12(50ng)	0.0003	***
	IKKα: EV vs. GRA12GRA12(100ng)	<0.0001	****
	IKKα: EV vs. GRA12GRA12(200ng)	<0.0001	****
Figure 4F	IKKβ: EV vs. GRA12GRA12(50ng)	0.0074	**
	IKKβ: EV vs. GRA12GRA12(100ng)	0.0011	**
	IKKβ: EV vs. GRA12GRA12(200ng)	0.0003	***
Figure 4G	p65: EV vs. GRA12(50ng)	0.0059	**
	p65: EV vs. GRA12(100ng)	0.0031	**
	p65: EV vs. GRA12(150ng)	0.0020	**
